# Synthetic Chalcones with Potent Antioxidant Ability on H_2_O_2_-Induced Apoptosis in PC12 Cells

**DOI:** 10.3390/ijms151018525

**Published:** 2014-10-14

**Authors:** Jian-Zhang Wu, Chan-Chan Cheng, Lai-Lai Shen, Zhan-Kun Wang, Shou-Biao Wu, Wu-Lan Li, Su-Hua Chen, Rong-Ping Zhou, Pei-Hong Qiu

**Affiliations:** 1 Chemical Biology Research Center, School of Pharmaceutical Sciences, Wenzhou Medical University, Wenzhou 325035, China; E-Mails: 15990761519@163.com (C.-C.C.); shenlailai@126.com (L.-L.S.); wushoubiao@163.com (S.-B.W.); honeybee13131@163.com (S.-H.C.); zrpbarra@163.com (R.-P.Z.); 2 Institute of Sports Science, Wenzhou Medical University, Wenzhou 325035, China; E-Mail: wzk2001@163.com; 3 College of Information Science and Computer Engineering, Wenzhou Medical University, Wenzhou 325035, China; E-Mail: liwulan@163.com

**Keywords:** chalcone derivatives, antioxidant, PC12 cells, Nrf2-ARE pathway, GCLC, HO-1

## Abstract

Chalcone derivatives (*E*)-3-(4-hydroxy-3-methoxyphenyl)-1-(4-methoxyphenyl) prop-2-en-1-one and (*E*)-3-(4-hydroxyphenyl)-1-(4-methoxyphenyl) prop-2-en-1-one (Compounds **1** and **2**) have been demonstrated to be potent anti-inflammatory agents in our previous study. In light of the relationship of intracellular mechanisms between anti-inflammatories and antioxidants, we further designed and synthesized a series of chalcone derivatives based on **1** and **2**, to explore their antioxidant efficacy. The majority of the derivatives exhibited strong protective effects on PC12 (PC12 rat pheochromocytoma) cells exposed to H_2_O_2_, and all compounds were nontoxic. A preliminary structure-activity relationship was proposed. Compounds **1** and **1d** ((*E*)-2-methoxy-4-(3-(4-methoxyphenyl)-3-oxoprop-1-en-1-yl) phenyl acrylate) exerted the action in a good dose-dependent manner. Quantitative RT-PCR (qRT-PCR) and western blot analysis showed that **1** and **1d** significantly improve the expression of nuclear factor erythroid 2 p45-related factor 2 (Nrf2)-dependent antioxidant genes *g-Glutamylcysteine Ligase Catalytic Subunit* (*GCLC*) and *heme oxygenase-1* (*HO-1*) and their corresponding proteins (γ-glutamyl cysteine synthase (γ-GCS) and HO-1) in PC12 cells. Inhibition of GCLC and HO-1 by specific inhibitors, l-buthionine-*S*-sulfoximine (BSO) and zinc protoporphyrin (ZnPP), respectively, partially reduce the protective effect of **1** and **1d**. These data present a series of novel chalcone analogs, especially compounds **1** and **1d**, as candidates for treating oxidative stress-related disease by activating the Nrf2-antioxidant responsive element (ARE) pathway.

## 1. Introduction

Oxidative stress is produced by reactive oxygen species (ROS), which can destroy the physiological function of cellular proteins, lipids, nucleic acids, and other macromolecular substances. Oxidative stress leads to aging and many human diseases including neurodegenerative disorders, such as Parkinson’s and Alzheimer’s diseases [1]. Upon the stimulation of ROS, a series of protective proteins, such as heme oxygenase-1 (HO-1), γ-glutamyl cysteine synthase (γ-GCS), superoxide dismutase, and NAD(P)H/quinone oxidoreductase 1 (NQO1), will be translated to alleviate cell damage [2]. The transcription of antioxidant proteins is regulated by antioxidant responsive elements (AREs). Nuclear factor erythroid 2 p45-related factor 2 (Nrf2) is the activating factor of ARE, and the Nrf2-ARE pathway is currently the most important endogenous antioxidant signaling pathway. As a major component of ROS, H_2_O_2_ causes cell apoptosis by inducing lipid peroxidation and DNA damage. Therefore, outstanding ability to reduce cell apoptosis induced by H_2_O_2_ or potent Nrf2-ARE pathway activation represents promising antioxidant capacity to ameliorate oxidative stress-related diseases.

Chalcones (1,2-diphenyl-2-propen-1-ones), which exist in fruits, vegetables, spices, tea, and soy-based foodstuff, constitute an important group of natural products belonging to the flavonoid family. They continuously attract considerable interest because of their remarkable biological potential as anticancer [3,4,5], anti-inflammatory [6,7], antifungal [8], antibacterial [9], antioxidant [10,11,12], and antitumor agents [13]. In the past decades, an increasing number of naturally occurring chalcones were isolated from plants, and various studies on the biological activities were performed. Moreover, many synthetic chalcones with modified groups have emerged, laying the foundation of exploring and improving the biological activity of chalcones.

In our previous study, chalcone derivative **1** and another similar structure **2** exhibited promising anti-inflammatory activities [14]. Data from other previous studies also showed that chalcones with good anti-inflammatory activity usually simultaneously possess significant antioxidant effects [15,16,17,18,19]. Some molecules possess both antioxidant and anti-inflammatory efficacy partially due to the capacity of inducing HO-1 expression, for HO-1 is a potent cytoprotective enzyme promptly induced by cells in response to severe oxidative stress as well as inflammatory reactions. Similar correlations in the molecular mechanisms may exist between antioxidant and anti-inflammatory activities triggered by chalcones. In the present study, we thus hypothesized that compounds **1** and **2** also possess antioxidant activities. Therefore a set of novel chalcones with acyl groups based on **1** and **2** were further synthesized for antioxidant screening. To date, there is no report concerning the antioxidant activity induced by acylated chalcones currently exists. To obtain a primary structure-activity relationship (SAR) and find novel chalcones with antioxidant ability, biological experiments were performed to screen for potent synthetic chalcones against H_2_O_2_-induced apoptosis in PC12 cells, a neuronal cell model broadly used in antioxidant studies [20,21,22]. This paper presents a series of novel chalcone analogs, especially compounds **1** and **1d**, as excellent Nrf2-ARE pathway activators.

## 2. Results and Discussion

### 2.1. Chemistry

**Scheme 1:**
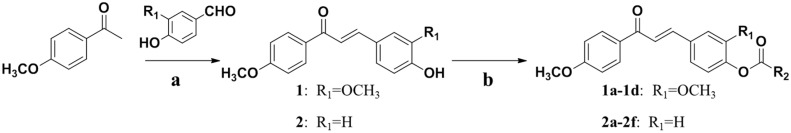
Formation of chalcone derivatives. Reagents and conditions: (**a**) Alcohol, concentrated sulfuric acid, reflex; and (**b**) Tetrahydrofuran, pyridine, room temperature.

The class **1** chalcone derivatives can be synthesized via the Claisen–Schmidt condensation of 4-methoxyacetophenone and 4-hydroxybenzaldehyde or 4-hydroxy-3-methoxybenzaldehyde in acidic conditions (Scheme 1). The substituted benzaldehyde and acetophenone were dissolved in ethanol and stirred at 78 °C, and added with a catalytic amount of concentrated sulfuric acid. The products precipitated at 4 °C after the reaction. Class **1** compounds contain only two chalcones, which were also used as reagents to synthesize class **2** chalcone derivatives. Class **2** compounds were produced at room temperature by the base-catalyzed nucleophilic substitution reaction. Tetrahydrofuran was used as a solvent and pyridine as a catalyst. The corresponding chalcone derivatives of class **2** (Table 1) were isolated by crystallization or silica gel chromatography. The end products were characterized by electrospray ionization mass spectrometry (ESI-MS), electrospray ionization high-resolution mass spectrometry (ESI-HRMS), ^1^H-NMR, ^13^C-NMR and elemental analysis. Compounds **1** and **2** were reported in our previous published articles, and chalcone **2b** ((*E*)-4-(3-(4-methoxyphenyl)-3-oxoprop-1-en-1-yl)phenyl propionate) was reported in other study [23], whereas others are new compounds whose melting point, yield, spectral data and elemental analysis are described in the Experimental Section.

### 2.2. Effects of Chalcones on the Viability of PC12 Cells Exposed to H_2_O_2_

**Figure 1:**
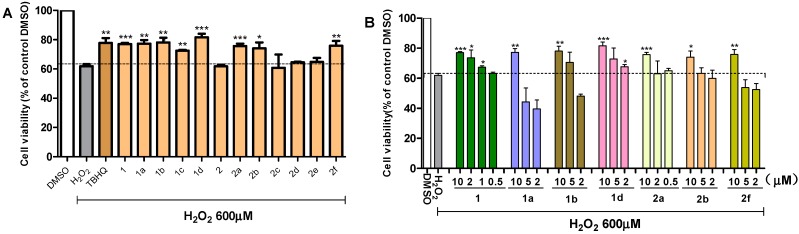
Chalcone derivatives protected PC12 cells from H_2_O_2_-induced oxidative injury. PC12 cells were seeded in a 96-well plate and pretreated with or without compounds for 24 h, and incubated in the presence or absence of H_2_O_2_ (600 µM) for 24 h. Control cells were only treated with medium containing 0.1% dimethyl sulfoxide (DMSO). (**A**) Screening of 12 chalcones at 10 μM, and TBHQ (*tert*-butylhydroquinone) (10 µM) served as a positive control; and (**B**) Dose-dependent evaluation of active compounds at concentrations ranging from 0.5 to 10 μM. Each bar represents mean ± SD of three to five independent experiments. Statistical significance relative to H_2_O_2_ group was expressed as follows: * *p* < 0.05, ** *p* < 0.01, *** *p* < 0.001.

All the synthetic chalcones were assessed for their protective effects against H_2_O_2_-induced cell death of PC12 cells by 3-(4,5-dimethylthiazol-2-yl)-2, 5-diphenyltetrazolium bromide (MTT) assay. TBHQ (*tert*-butylhydroquinone), a well-known potent antioxidant agent, was included as a positive control. As shown in Figure 1A, the majority of the chalcones (10 μM) presented significant ability to elevate viability of PC12 cells challenged with 600 µM H_2_O_2_. Among them, compounds **1**, **1a**, **1b**, **1d**, **2a**, **2b** and **2f** showed a similar effect of positive control. The positive role of methoxyl group at meta-position can be clearly seen by comparing chalcones **1** and **1c** to their unsubstituted analogues (**2**, **2c**). Among all the acylation products (class **2** compounds), the compounds with a chlorobenzene group (**1c**, **2c**) were found to be relatively low activity, while compounds with a short fatty chain of acyl group (**1a**, **1b**, **1d**, **2a**, **2b**) all exhibited good effect, and **1d** was the most effective. The above analysis revealed that electron-donating substituents in the benzene rings may be always favorable to the antioxidant efficiency. To note that, chalcone derivative **2f** with a fluorobenzene group was also active, indicating that the antioxidant actions of electro-withdrawing groups on the aromatic ring is still confused and requires further investigation. In general, all the acylation products showed good or increased antioxidant ability, suggesting that acylation may be a feasible measure to improve the antioxidant activity of 4-hydroxy chalcones.

**Table 1:** Structure of synthesized chalcone derivatives.

Compound	R_2_	Compound	R_2_
**1a**	CH_3_–	**2b**	CH_3_CH_2_–
**1b**	CH_3_CH_2_–	**2c**	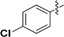
**1c**	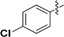	**2d**	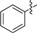
**1d**	CH_2_=CH–	**2e**	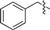
**2a**	CH_3_–	**2f**	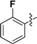

**Figure 2:**
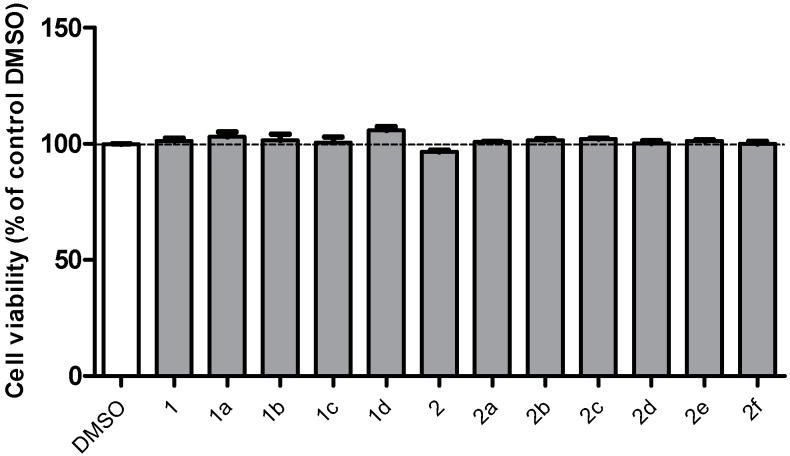
Cell viability assay shows that the compounds were nontoxic.

Concurrent with cell viability analysis, the cytotoxicity of the compounds (10 µM) was also tested using MTT. All the derivatives were nontoxic (Figure 2).

### 2.3. Active Compounds Dose-Dependently Increase the Viability of PC12 Cells Exposed to H_2_O_2_

All the aforementioned active compounds were tested for their ability to enhance cell viability in various concentrations ranging from 0.5 to 10 μM. Concentration gradients of all the active compounds differed. Cells were pretreated with different derivatives for 24 h, and incubated with 600 µM H_2_O_2_ for another 24 h. The results are shown in Figure 1B. Compounds **1**, **1a**, **1b**, **1d**, **2b**, and **2f** exhibited dose-dependent activation, and **1** and **1d** showed better action gradients. Furthermore, compound **1** appears to be active even at 0.5 µM.

### 2.4. **1** and **1d** Effectively Attenuate H_2_O_2_-Induced Cell Apoptosis

**Figure 3:**
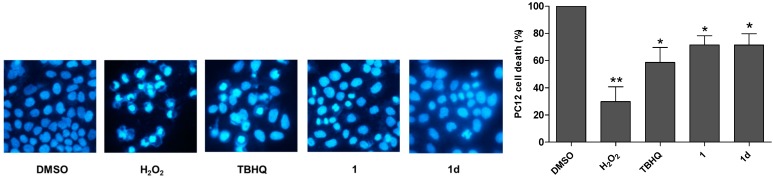
Chalcones **1** and **1d** attenuated H_2_O_2_-induced apoptosis of PC12 cells. Cell apoptosis was tested by the Hoechst staining assay (400×). Cells treated with DMSO alone or merely challenged with 600 µM H_2_O_2_ were used as the control. Compounds TBHQ, **1** and **1d** at 10 µM were pre-incubated in culture medium for 24 h, and then treated with 600 µM H_2_O_2_ for another 24 h. Values shown are the mean ± SD of five independent measurements. Statistical significance relative to DMSO group is indicated. * *p* < 0.05, ** *p* < 0.01.

**1** and **1d** were also examined the ability to protect against H_2_O_2_-triggered cell apoptosis using the Hoechst staining assay (400×). Apoptosis is morphologically characterized by nuclear condensation and segregation. As illustrated in Figure 3, cells exposed to 600 µM H_2_O_2_ demonstrated cracking, whereas cells cultured only with DMSO were integral. After pretreatment with **1** or **1d** at 10 µM, PC12 cells were better protected compared to TBHQ, which underscores the marked antioxidant ability of **1** and **1d**.

### 2.5. Active Compounds Significantly Elevate the Expression of Antioxidant Genes GCLC and HO-1

ARE-regulated genes, namely, NQO1, GCLC, g-Glutamylcysteine Ligase Modulatory Subunit (GCLM), and HO-1, constitute four main phase II detoxification genes. The transcriptional induction of these antioxidant genes through an ARE is largely dependent on Nrf2, which suggests that compounds may up-regulate antioxidant genes via Nrf2 activation. In this study, we selected seven relatively highly active compounds **1**, **1b**, **1c**, **1d**, **2a**, **2b**, and **2f**, according to the viability assay for further study. The mRNA levels of GCLC and HO-1 were detected to screen potent novel Nrf2-ARE inducers. PC12 cells were treated with chalcone derivatives (10 μM) for 6 h and the mRNA levels were examined by qRT-PCR. TBHQ was employed as a positive control.

**Figure 4:**
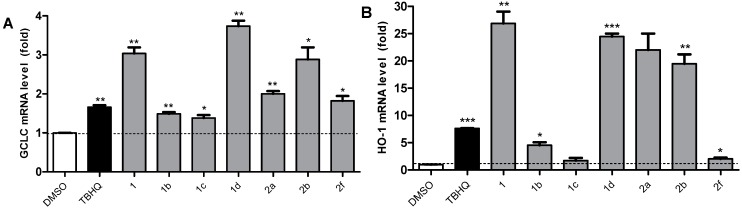
Seven active chalcones up-regulate the GCLC and HO-1 mRNA levels in PC12 cells. Cells were pretreated with 10 µM chalcones or vehicle control for 6 h. The mRNA levels of antioxidant genes GCLC (**A**) and HO-1 (**B**) were measured by qRT-PCR. The mRNA values for each gene were normalized to those of internal control GAPDH (glyceraldehyde phosphate dehydrogenase) mRNA, and expressed as a ratio to DMSO. Each bar represents the mean ± SD of three separate experiments. Statistical significance relative to DMSO group is indicated as follows: * *p* < 0.05, ** *p* < 0.01, *** *p* < 0.001.

The results of Nrf2-ARE enhancement evaluation are shown in Figure 4. All seven chalcone derivatives clearly increased the GCLC and HO-1 mRNA levels. For GCLC activation, compounds **1**, **1d**, and **2b** were more effective than the positive control TBHQ, and **1d** was the most effective. The expression of HO-1 showed more significant changes than that of GCLC. For HO-1 expression, four compounds (**1**, **2d**, **2a**, and **2b**) treated in PC12 cells showed outstanding effects. **1** was the best inducer, which was about 25-fold higher compared with the control group. Thus, compounds **1** and **1d** were the most potent activators. Among the four downstream genes of the Nrf2-ARE pathway, HO-1 is the most induced antioxidant gene in melanocytes challenged with H_2_O_2_ [24]. The data in this study further confirmed the crucial role of HO-1 in the resistance of oxidative stress in neuronal cells. Furthermore, the end products of HO-1, including biliverdin, carbon monoxide, and ferrous iron, also have potent antioxidant activities. Overall, HO-1 is a critical factor in the cytoprotective effects. Thus, we screened out **1** and **1d**, which showed the highest HO-1 and GCLC mRNA expression levels, as the two strongest activators of Nrf2-regulated antioxidant defenses.

### 2.6. **1** and **1d** Up-Regulate γ-GCS and HO-1 Protein Levels in PC12 Cells

Similar to HO-1, γ-GCS (also known as GCL (glutamate cysteine ligase)) is another phase II antioxidant enzyme composed of a catalytic subunit (GCLC) and regulatory subunit (GCLM), and is the rate-limiting enzyme for glutathione (GSH) synthesis. GSH is a tripeptide with diverse functions, including modulation of cell proliferation, antioxidant defense, and detoxification of xenobiotics [25]. To elucidate the mechanism of active compounds, western blot assays were used to determine the expression levels of two antioxidant proteins γ-GCS and HO-1 in PC12 cells. PC12 cells were treated with the most potent chalcones (*i.e.*, **1** and **1d**) at 0.5, 2, and 10 μM for 24 h. We selected compound **2**, which showed no activity, as the negative control, and GAPDH was used as the internal standard.

**Figure 5:**
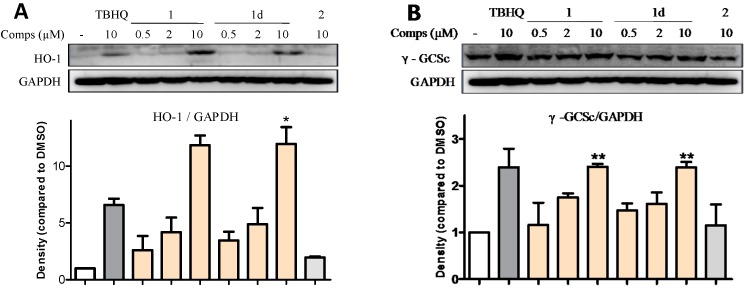
**1** and **1d** affected γ-GCS and HO-1 protein levels in PC12 cells. Cells were treated with **1** or **1d** at the concentrations of 0.5, 2, and 10 µM. Compound 2 was used as the negative control. (**A**) **1** and **1d** elevated the expression level of protein HO-1; and (**B**) **1** and **1d** heightened the intracellular content of protein γ-GCS. The increasing levels of HO-1 and γ-GCS were dose-dependent. GAPDH served as a loading control. Representative values are the mean ± SD of three independent experiments. * *p* < 0.05, ** *p* < 0.01, relative to cells treated only with DMSO.

As shown in Figure 5, compounds **1** and **1d** at 0.5 and 2 μM showed no obvious effect on the enhancement of proteins HO-1 and γ-GCS. However, these chalcones highly increased the intracellular contents of HO-1 and γ-GCS at 10 μM. The γ-GCS level triggered by these two chalcones was similar to that of TBHQ, whereas the HO-1 protein level was much higher than that of TBHQ. The results confirm that **1** and **1d** improved the cell viability in a concentration-dependent manner. In line with the data of qRT-PCR, **1** and **1d** exerted similar abilities to activate ARE-regulated genes HO-1 and GCLC.

### 2.7. Induction of HO-1 and GCLC Expression Is Responsible for the Antioxidant Activities of **1** and **1d**

**Figure 6:**
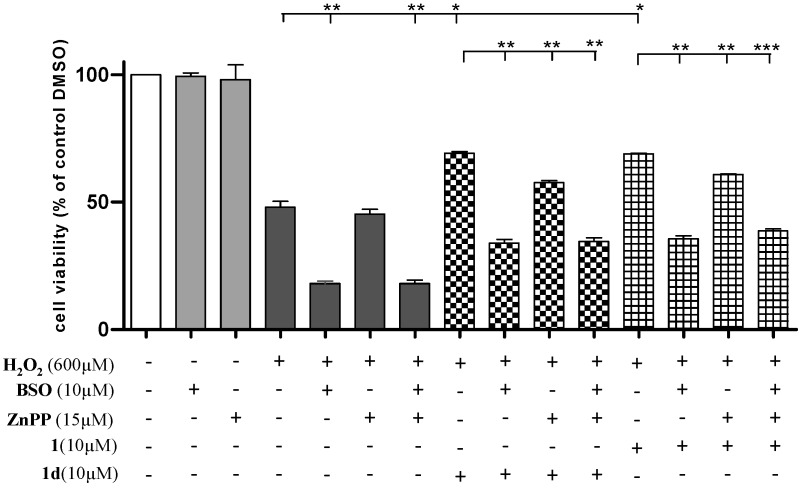
Protective effects of **1** and **1d** against H_2_O_2_-induced cell damage diminished after treatment of PC12 cells with ZnPP or BSO. ZnPP and BSO were dissolved in 100 mM NaOH solution and PBS, respectively. These two inhibitors were added into the culture medium 1 h prior to the addition of chalcones. Cells were pretreated with **1** and **1d** for 24 h before exposure to 600 µM H_2_O_2_ for an additional 24 h. The final concentrations of ZnPP and BSO were 15 and 10 µM, respectively. Representative data are the mean ± SD of three independent experiments, * *p* < 0.05, ** *p* < 0.01, *** *p* < 0.001, compared with the H_2_O_2_ group.

To investigate whether the increased expression of HO-1 and GCLC caused by the identified lead compounds **1** and **1d** is responsible for the cytoprotective effects against H_2_O_2_-derived oxidative cell death, specific inhibitors, ZnPP and BSO were utilized in this study. We treated ZnPP (15 µM) or BSO (10 µM) in PC12 cells 1 h before the addition of chalcones. Meanwhile, we also determined whether the concentrations of ZnPP and BSO used in this study cause cell damage. As shown in Figure 6, no significant adverse effect was observed on the viability of PC12 cells after cells were treated only with ZnPP or BSO (cell viability: 99.34% ± 2.60% and 98.07% ± 0.80%, respectively). In the groups without chalcone treatment, intracellular GCLC and HO-1 production triggered by H_2_O_2_ protected PC12 cells from apoptosis, whereas chalcone treatment enhanced the protective effects of PC12 cells challenged with H_2_O_2_. The result showed a dramatic decrease in cell viability induced by chalcones after BSO was applied. The beneficial function of HO-1 caused by chalcones was easily found by comparing the groups that included ZnPP to their untreated controls. When both GCLC and HO-1 were inhibited, the group showed similar low cell viability to the group in which only GCLC was resisted, thereby indicating that the generation of HO-1 occurred in association with the intracellular GCLC content. Given that GSH mediated the procedure of HO-1 expression [26], as a key element of GSH synthesis, GCLC indirectly regulated the generation of HO-1. These results suggest that the induction of HO-1 and GCLC expression had a crucial function in suppressing oxidative stress, and partially explain the antioxidant activity of **1** and **1d**.

## 3. Experimental Section

### 3.1. Chemical Synthesis

All chemical reagents were commercial products purchased from Sigma–Aldrich, Fluka, and Aladdin (Beijing, China) and used without further purification. All reactions were monitored by thin layer chromatography (TLC) with silica gel GF254 and observed under UV light. Silica gel (200 to 300 mesh) was obtained from Qingdao Haiyang Chemical Ltd. (Shandong, China) for column chromatography. Melting points were tested in open capillary tubes on a Fisher–Johns melting apparatus. Low-resolution mass spectra were performed on a Bruker Esquire HCT spectrometer and High-resolution mass spectra were recorded on a Bruker micrOTOF-QII spectrometer, respectively. The ^1^H-NMR and ^13^C-NMR spectra were obtained on a 600 MHz apparatus (Bruker Corporation, Ettlingen, Germany) using trimethylsilyl (TMS) as an internal standard. The splitting patterns were described as follows: s = singlet, d = doublet, t = triplet, q = quartet, dd = doublet of doublets, m = multiplet.

For the reactions, 4-methoxyacetophenone (20 mmol) and 4-hydroxybenzaldehyde (20 mmol) or 4-hydroxy-3-methoxybenzaldehyde (20 mmol) were dissolved in ethanol (40 mL). The mixture was stirred at 78 °C and added with 0.5 mL of concentrated sulfuric acid. After 6 h, the reaction liquid was cooled in 4 °C. The products of **1** or **2** precipitated, and filtered off for the preparation of class **2** compounds. In the next step, corresponding acyl chloride (3 mmol, excess) was added to 12 mL of tetrahydrofuran, which dissolved 2 mmol **1** or **2**. Approximately 0.5 mL of pyridine was used as a catalyst. The reaction mixture was then stirred at room temperature and monitored by TLC. Water was added into the reaction mixture after 24 h to precipitate the crude product, and 5% ethanol/water was used to wash the product. The crude products of **1**, **1d**, and **2f** were purified by crystallization, and the other compounds were purified by column chromatography using PE/EA as the solvent system in increasing order of polarity. The chemical data of novel compounds are presented as follows:

2-Methoxy-4-((*E*)-3-(4-methoxyphenyl)-3-oxoprop-1-enyl) phenyl acetate (**1a**): Light yellow powder, 76.5% yield; mp (melting point) 106.9–109.2 °C; ^1^H-NMR (CDCl_3_, 600 MHz) δ: 8.033 (d, *J* = 9 Hz, 2H, H-2', H-6'), 7.748 (d, *J* = 15.6 Hz, 1H, H-β), 7.465 (d, *J* = 15.6 Hz, 1H, H-α), 7.250 (dd, *J* = 1.8 Hz, 7.8 Hz, 1H, H-6), 7.196 (d, *J* = 1.8 Hz, 1H, H-2), 7.084 (d, *J* = 7.8 Hz, 1H, H-5), 6.990 (d, *J* = 8.4 Hz, 2H, H-3', H-5'), 3.900 (s, 3H, OCH_3_-4'), 3.895 (s, 3H, OCH_3_-3), 2.334 (s, 3H, CH_3_-4); ^13^C-NMR (CDCl_3_, 600 MHz) δ: 188.76 (C=O), 168.93 (Ar–O–C=O), 163.63, 151.57, 143.44, 141.61, 134.24, 131.18, 130.98 (×2), 123.44, 122.33, 114.02 (×2), 112.00, 56.13 (OCH_3_), 55.65 (OCH_3_), 20.08 (CH_3_); ESI-MS *m*/*z*: 327.3 [M + H]^+^; ESI-HRMS *m*/*z* [M + H]^+^ calculated for C_19_H_18_O_5_: 327.1232, found: 327.1231; Anal (elementary analysis). calculated for C_19_H_18_O_5_: C 69.93, H 5.56, found: C 69.84, H 5.57.

2-Methoxy-4-((*E*)-3-(4-methoxyphenyl)-3-oxoprop-1-enyl) phenyl propionate (**1b**): Yellow powder, 96.07% yield; mp 117.3-119.5 °C; ^1^H-NMR (CDCl_3_, 600 MHz) δ: 8.035 (d, *J* = 8.4 Hz, 2H, H-2', H-6'), 7.750 (d, *J* = 15.6 Hz, 1H, H-β), 7.465 (d, *J* = 15.6 Hz, 1H, H-α), 7.251 (dd, *J* = 1.8 Hz, 8.4 Hz, 1H, H-6), 7.192 (s, *J* = 1.8 Hz, 1H, H-2), 7.079 (d, *J* = 8.4 Hz, 1H, H-5), 6.990 (d, *J* = 9.0 Hz, 2H, H-3', H-5'), 3.896 (s, 3H, OCH_3_-3), 3.892 (s, 3H, OCH_3_-4'), 2.635 (q, *J* = 7.8 Hz, 2H, COCH_2_), 1.288 (t, *J* = 7.2 Hz, 3H, CH_3_); ^13^C-NMR (CDCl_3_, 600 MHz) δ: 188.78 (C=O), 172.47 (Ar–C=O), 163.62, 151.62, 143.50, 141.77, 134.10, 131.19, 130.98 (×2), 114.02 (×2), 56.15 (OCH_3_), 55.64 (OCH_3_), 27.52 (CH_2_), 9.28 (CH_3_); ESI-MS *m*/*z*: 341.3 [M + H]^+^; ESI-HRMS *m*/*z* [M + H]^+^ calculated for C_20_H_20_O_5_: 341.1389, found 341.1384; Anal. calculated for C_20_H_20_O_5_: C 70.58, H 6.05, found: C 70.58, H 6.05.

2-Methoxy-4-((*E*)-3-(4-methoxyphenyl)-3-oxoprop-1-enyl) phenyl 4-chlorobenzoate (**1c**): Light yellow powder, 91.2% yield; mp 254.5–255.7 °C; ^1^H-NMR (CDCl_3_, 600 MHz) δ: 8.154 (d, *J* = 8.4 Hz, 2H, CO-Ar-H^2^, CO-Ar-H^6^), 8.049 (d, *J* = 9 Hz, 2H, H-2', H-6'), 7.783 (d, *J* = 15.6 Hz, 1H, H-β), 7.499 (d, *J* = 15.6 Hz, 1H, H-α), 7.498 (d, *J* = 8.4 Hz, 2H, CO–Ar–H^3^, CO–Ar–H^5^), 7.306 (dd, *J* = 1.8 Hz, *J* = 8.4 Hz, 1H, H-6), 7.244 (d, *J* = 1.8 Hz, 1H, H-2), 7.202 (d, *J* = 8.4 Hz, 1H, H-5), 6.999(d, *J* = 9 Hz, 2H, H-3', H-5'), 3.900 (s, 3H, OCH_3_-3), 3.885 (s, 3H, OCH_3_-4'); ESI–MS *m*/*z*: 423.0 [M + H]^+^; ESI–HRMS *m*/*z* [M +·Na]^+^ calcd for C_24_H_19_ClO_5_: 445.0818, found 445.0813; Anal. calculated for C_24_H_19_ClO_5_: C 68.17, H 4.53, found: C 68.08, H 4.37.

2-Methoxy-4-((*E*)-3-(4-methoxyphenyl)-3-oxoprop-1-enyl) phenyl acrylate (**1d**): Light yellow crystal, 20.22% yield; mp 157.9–160.9 °C; ^1^H-NMR (CDCl_3_, 600 MHz) δ: 8.037 (d, *J* = 9 Hz, 2H, H-2', H-6'), 7.757 (d, *J* = 15.6 Hz, 1H, H-β), 7.475 (d, *J* = 15.6 Hz, 1H, H-α), 7.269 (dd, *J* = 1.8 Hz, 7.8 Hz, 1H, H-6), 7.212 (d, *J* = 1.2 Hz, 1H, H-2), 7.127 (d, *J* = 7.8 Hz, 1H, H-5), 6.992 (d, *J* = 9 Hz, 2H, H-3', H-5'), 6.632 (d, *J*_trans_ = 17.4 Hz, 1H, CO–C=CH), 6.360 (dd, *J*_cis_ = 10.2 Hz, *J*_trans_ = 17.4 Hz), 6.040 (d, *J*_cis_ =10.8 Hz, 1H, CO–C=CH), 3.894 (s, 6H, OCH_3_-3, OCH3-4'); ^13^C-NMR (CDCl_3_, 600 MHz) δ: 188.79 (C=O), 164.00 (Ar–C=O), 163.64, 151.63, 143.45, 141.45, 134.28 (O=C–CH=CH_2_), 133.07, 131.17, 130.99 (×2), 127.52 (O=C–CH=CH_2_), 123.43, 122.35, 121.36, 114.03 (×2), 112.09, 56.17 (OCH_3_), 55.65 (OCH_3_); ESI-MS *m*/*z:* 339.3 [M + H]^+^; ESI-HRMS *m*/*z* [M + H]^+^ calculated for C_20_H_18_O_5_: 339.1233, found: 339.1228; Anal. calculated for C_20_H_18_O_5_: C 70.99, H 5.36, found: C 70.81, H 5.19.

4-((*E*)-3-(4-Methoxyphenyl)-3-oxoprop-1-enyl) phenyl acetate (**2a**): White powder, 90.0% yield; mp 137.2–138.2 °C; ^1^H-NMR (CDCl_3_, 600 MHz) δ: 8.035 (d, *J* = 9 Hz, 2H, H-2', H-6'), 7.778 (d, *J* = 15.6 Hz, 1H, H-β), 7.657 (d, *J* = 8.4 Hz, 2H, H-2, H-6), 7.499 (d, *J* = 15 Hz, 1H, H-α), 7.156 (d, *J* = 8.4 Hz, 2H, H-3, H-5), 6.987 (d, *J* = 9 Hz, 2H, H-3', H-5'), 3.893 (s, 3H, OCH_3_-4'), 2.322 (s, 3H, OCOCH_3_-4); ^13^C-NMR (CDCl_3_, 600 MHz) δ: 188.68 (C=O), 169.28 (Ar-C=O), 163.63, 152.27, 142.96, 132.98, 131.51, 131.19, 130.96, 130.79 (×2), 129.61 (×2), 127.22, 122.30 (×2), 122.18, 121.52, 114.02 (×2), 55.64 (OCH_3_), 21.28 (OCH_3_). ESI-MS *m*/*z*: 297.1 [M + H]^+^; ESI-HRMS *m*/*z* [M + H]^+^ calculated for C_18_H_16_O_4_: 297.1127, found: 297.1121; Anal. calculated for C_18_H_16_O_4_: C 72.96, H 5.44, found: C 72.99, H 5.52.

4-((*E*)-3-(4-Methoxyphenyl)-3-oxoprop-1-enyl) phenyl propionate (**2b**): Light yellow powder, 72.95% yield; mp 114.3–114.9 °C; ^1^H-NMR (CDCl_3_, 600 MHz) δ: 8.037 (d, *J* = 7.2 Hz, 2H, H-2', H-6'), 7.781 (d, *J* = 15.0 Hz, 1H, H-β), 7.655 (d, *J* = 8.4 Hz, 2H, H-2, H-6), 7.499 (d, *J* = 15.6 Hz, 1H, H-α), 7.154 (d, *J* = 6.6 Hz, 2H, H-3, H-5), 6.987 (d, *J* = 6.9 Hz, 2H, H-3', H-5'), 3.894 (s, 3H, OCH_3_-4'), 2.614 (q, *J* = 7.2 Hz, 2H, OCOCH_2_-4), 1.280 (t, *J* = 7.2 Hz, 3H, CH_3_); ESI-MS *m*/*z*: 311.0 [M + H]^+^; ESI–HRMS *m*/*z* [M + H]^+^ calculated for C_19_H_18_O_4_: 311.1283, found: 311.1287; Anal. calculated for C_19_H_18_O_4_: C 73.53, H 5.85, found: C 73.40, H 5.91.

4-((*E*)-3-(4-Methoxyphenyl)-3-oxoprop-1-enyl) phenyl 4-chlorobenzoate (**2c**): Yellow powder, 67.5% yield; mp 162.5–164 °C; ^1^H-NMR (CDCl_3_, 600 MHz) δ: 8.145 (d, *J* = 9 Hz, 2H, CO-Ar-H^2^, CO–Ar–H^6^), 8.048 (d, *J* = 8.4 Hz, 2H, H-2', H-6'), 7.812 (d, *J* = 15.6 Hz, 1H, H-β), 7.714 (d, *J* = 8.4 Hz, 2H, H-2, H-6), 7.532 (d, *J* = 15.6 Hz, 1H, H-α), 7.504 (d, *J* = 8.4 Hz, 2H, CO–Ar–H^3^, CO–Ar–H^5^), 7.281 (d, *J* = 8.4 Hz, 2H, H-3, H-5), 6.994 (d, *J* = 8.4 Hz, 2H, H-3', H-5'), 3.898 (s, 3H, OCH_3_-4'); ESI–MS *m*/*z*: 393.3 [M + H]^+^; ESI–HRMS *m*/*z* [M + H]^+^ calculated for C_23_H_17_ClO_4_: 393.0894, found: 393.0899; Anal. calculated for C_23_H_17_ClO_4_: C 70.32, H 4.36, found: C 70.33, H 4.25.

4-((*E*)-3-(4-Methoxyphenyl)-3-oxoprop-1-enyl) phenyl benzoate (**2d**): Yellow powder, 95.9% yield; mp 138.9–140.2 °C; ^1^H-NMR (CDCl_3_, 600 MHz) δ: 8.214 (d, *J* = 7.8 Hz, 2H, CO–Ar–H^2^, CO–Ar–H^6^), 8.052 (d, *J* = 9.0 Hz, 2H, H-2', H-6'), 7.816 (d, *J* = 15.6 Hz, 1H, H-β), 7.716 (d, *J* = 9.0 Hz, 2H, H-2, H-6), 7.658 (t, *J* = 7.8 Hz, 1H, CO–Ar–H^4^), 7.536 (d, *J* = 8.4 Hz, 2H, H-3, H-5), 7.533 (d, *J* = 15.6 Hz, 1H, H-α), 7.295 (d, *J* = 8.4 Hz, 2H, CO–Ar–H^3^, CO–Ar–H^5^), 6.995 (d, *J* = 9.0 Hz, 2H, H-3, H-5), 3.898 (s, 3H, OCH_3_-4'); ^13^C-NMR (CDCl_3_, 600 MHz) δ: 188.72 (C=O), 165.05 (Ar–C=O), 163.64, 152.60, 143.03, 133.96, 133.06, 131.22, 130.99 (×2), 130.38 (×2), 129.69 (×2), 129.41, 128.80 (×2), 122.47 (×2), 122.21, 114.04 (×2), 55.66 (OCH_3_); ESI–MS *m*/*z*: 359.3 [M + H]^+^; ESI-HRMS *m*/*z* [M + H]^+^ calculated for C_23_H_18_O_4_: 359.1283, found: 359.1278; Anal. calculated for C_23_H_18_O_4_: C 77.80, H 5.06, found: C 77.69, H 5.12.

4-((*E*)-3-(4-Methoxyphenyl)-3-oxoprop-1-enyl) phenyl 2-phenylacetate (**2e**): Yellow powder, 56.5% yield; mp 102.3–106 °C; ^1^H-NMR (CDCl_3_, 600 MHz) δ: 8.029 (dd, *J* = 1.8 Hz, 7.2 Hz, 2H, H-2', H-6'), 7.761 (d, *J* = 15.6 Hz, 1H, H-β), 7.631 (d, *J* = 8.4 Hz, 2H, H-2, H-6), 7.485 (d, *J* = 15.6 Hz, 1H, H-α), 7.379–7.390 (m, 3H, CO–C–Ar–H^2,4,6^), 7.302–7.325 (m, 2H, CO–C–Ar–H^3,5^), 7.129 (d, *J* = 8.4 Hz, 2H, H-3, H-5), 6.983 (dd, *J* = 1.8 Hz, 7.2 Hz, 2H, H-3', H-5'), 3.890 (s, 3H, OCH_3_-4'), 3.879 (s, 2H, CO–CH_2_–Ar); ESI–MS *m*/*z*: 373.2 [M + H]^+^; ESI–HRMS *m*/*z* [M + Na]^+^ calculated for C_24_H_20_O_4_: 395.1259, found: 395.1254; Anal. calculated for C_24_H_20_O_4_: C 77.40, H 5.41, found: C 77.19, H 5.38.

4-((*E*)-3-(4-Methoxyphenyl)-3-oxoprop-1-enyl) phenyl 2-fluorobenzoate (**2f**): Yellow crystal, 47.99% yield; mp 150.4–157.1 °C; ^1^H-NMR (Acetone-*d*_6_, 600 MHz) δ: 8.114 (td, *J* = 1.2 Hz, 7.5 Hz, 1H, CO–Ar–H^6^), 8.053 (d, *J* = 9 Hz, 2H, H-2', H-6'), 7.815 (d, *J* = 15.6 Hz, 1H, H-β), 7.716 (d, *J* = 9 Hz, 2H, H-2, H-6), 7.535 (d, *J* = 15.6 Hz, 1H, H-α), 7.311 (d, *J* = 8.4 Hz, 2H, H-3, H-5), 7.294 (t, *J* = 7.2 Hz, 1H, CO–Ar–H^4^), 7.159–7.244 (m, 2H, CO–Ar–H^3^, CO–Ar–H^5^), 6.996 (d, *J* = 8.4 Hz, 2H, H-3', H-5'), 3.899 (s, 3H, OCH_3_-4'); ESI–MS *m*/*z*: 377.1 [M + H]^+^; ESI–HRMS *m*/*z* [M + Na]^+^ calculated for C_23_H_17_FO_4 _: 399.1009, found: 399.1009; Anal. calculated for C_23_H_17_FO_4_: C 73.40, H 4.55, found: C 73.35, H 4.56.

### 3.2. Biology

#### 3.2.1. Reagents and Cell Culture

MTT, ZnPP, BSO, and chemical reagents were obtained from Sigma (Louis, MO, USA). H_2_O_2_ was obtained from Sinopharm Chemical (Shanghai, China). DMSO was purchased from Sijia Biotechnology (Guangzhou, China). Hoechst Staining Kit was obtained from Beyotime Institute of Biotechnology (Shanghai, China). Reverse transcription and RT-PCR were carried out using a M-MLV first strand kit (Invitrogen, Carlsbad, CA, USA) and iQ SYBR Green Supermix kit (Bio-Rad Laboratories, Singapore), respectively. The γ-GCSc and HO-1 antibodies were purchased from Santa Cruz Biotechnology (Santa Cruz). Rat PC12 cells were obtained from the Institute of Biochemistry and Cell Biology, SIBS, CAS. PC12 cells were cultured in DMEM medium (Gibco, Beijing, China) (pH 7.4) supplemented with 10% (*v*/*v*) fetal bovine serum (Hyclone, Logan, UT, USA) and antibiotics (100 units/mL penicillin, 100 μg/mL streptomycin) at 37 °C in a 5% CO_2_ humidified incubator. Chalcone derivatives were dissolved in DMSO (analytical grade), and the DMSO concentration in the cell culture medium did not exceed 0.1%.

#### 3.2.2. Cell Viability Assay

Hydrogen peroxide solution was freshly prepared in PBS to a required concentration. Compounds were dissolved in DMSO (2 × 10^−2^ M) for storing, and diluted in cell culture medium before adding to a 96-well plate (4 × 10^3^ cells per well). The final concentration of the chalcones or TBHQ was 10 μM, and that of DMSO was 0.1%. After 24 h of pre-incubation, H_2_O_2_ at 600 μM was added for an additional 24 h. Approximately 20 μL of MTT solution (5 mg/mL) was added to each well after H_2_O_2_ challenge, and then incubated for another 4 h. MTT and cell culture liquid were removed, and 120 μL of DMSO was added to dissolve the purple formazan crystals. The absorbance at 570 nm was read in a microplate reader. The absorbance of the sample treated with 0.1% DMSO alone was regarded as 100% cell viability.

#### 3.2.3. Hoechst Staining

The fluorescent dye Hoechst is widely used in detecting the nuclear fragmentation of apoptotic cells. PC12 cells were seeded in a six-well plate and pretreated with **1** or **1d** for 24 h. The cells were then challenged with 600 μM H_2_O_2_ for an additional 24 h. Apoptotic cells were stained according to the Hoechst staining kit (Beyotime Institute of Biotechnology, Shanghai, China), and plates were observed under a fluorescence microscope.

#### 3.2.4. RNA Extraction and qRT-PCR

The cell culture medium was removed from culture plate, and cells were washed twice with cold PBS. Approximately 1 mL of TRIzol (Invitrogen, Carlsbad, CA, USA) was added to extract the total RNA of PC12 cells. Each cell sample was mixed with chloroform and centrifuged. The supernatant was collected and mixed with an equal volume of isopropyl alcohol. The supernatant was discarded after centrifugation. The RNA precipitate was gently rinsed by 75% ethanol and centrifuged. DEPC (diethyl pyrocarbonate, 0.1% water solution) was added to dissolve the RNA precipitate, and the RNA concentration was detected on a microplate reader. Reverse transcription and amplification of target genes *GCLC* and *HO-1*, as well as the internal control *GAPDH*, were performed according to the manufacturer’s instructions (Invitrogen, Carlsbad, CA, USA). The synthetic double-stranded *HO-1*, *GCLC*, and internal standard *GAPDH* oligonucleotide sequence were as follows: HO-1 (forward: GCCTGCTAGCCTGGTTCAAG; reverse: AGCGGTGTCTGGGATGAACTA), GCLC (forward: GTCCTCAGGTGACATTCCAAGC; reverse: TGTTCTTCAGGGGCTCCAGTC), and GAPDH (forward: AAGCTGGTCATCAACGGGAAAC; reverse: GAAGACGCCAGTAGACTCCACG). Amplification of HO-1, GCLC, and GAPDH was performed by heating samples to 95 °C for 2 min, and samples were subjected to 40 cycles of denaturation for 15 s at 95 °C and annealing for 30 s at 60 °C.

#### 3.2.5. Western Blot Analysis

After pretreatment with chalcones for 24 h, the cell medium was removed and PC12 cells were washed twice with PBS. The cells were then lysed with lysis buffer. Proteins were extracted from PC12 cells and quantified using a BCA Protein Assay kit (Institute of Biotechnology, Shanghai, China). Equal amounts of protein were separated by 10% SDS-polyacrylamide gel, and transferred onto PVDF membranes (Reanta, Beijing, China). The membrane was blocked with nonfat milk for 2 h, and incubated overnight with primary antibody of γ-GCS (1:1000, Santa Cruz) or HO-1 (1:1000, Santa Cruz) at 4°C. After three washes with TBST (Tris-buffered saline with 0.1% Tween-20, Sijia biological technology Co., LTD, Guangzhou, China), the blots were incubated with horseradish-peroxidase-conjugated secondary antibody in TBST at a 1:3000 dilution for 1 h at room temperature. Antibody-bound proteins were detected using enhanced chemiluminescence detection, and the film was exposed. GAPDH served as an internal control.

## 4. Conclusions

In summary, we synthesized a series of novel chalcone analogues and identified **1** and **1d** as the two strongest agents to alleviate H_2_O_2_-induced cell injury. Compounds **1**, **1a**, **1b**, **1d**, **2a**, **2b**, and **2f** improved the viability of PC12 cells in a dose-dependent manner. The results of qRT-PCR and western blot analysis show that these compounds protected against oxidative stress-induced neuronal cell death because of their preconditioning effect on Nrf2-ARE activation. In the analysis of the SAR, the methoxyl group at the meta-position was favorable for the protective effect. Moreover, the antioxidant activity of 4-hydroxychalcones may be prompted after acylation. This study provides additional information on the structural features that increase antioxidant efficiency. However, the underlying molecular mechanisms have yet to be elucidated, and further studies are necessary to develop compounds **1** and **1d** as potential candidates for oxidative stress-related diseases.
